# Impact of bariatric surgery on metabolic-dysfunction associated fatty liver disease in diabetic and non-diabetic patients: a cohort study

**DOI:** 10.1186/s12876-025-04524-4

**Published:** 2025-12-07

**Authors:** Mohammad Sistanizad, Niloufar Taherpour, Peyman Alibeigi, Soheila Sadeghi, Mojan Shamizadehkalkhoran, Omid Moradi

**Affiliations:** 1 Prevention of Cardiovascular Disease Research Center, Imam Hossein Hospital, Shahid Beheshti University of Medical Sciences, Tehran, Iran; 2 Department of Clinical Pharmacy, School of Pharmacy, Shahid Beheshti University of Medical Sciences, Tehran, Iran; 3 Prevention of Cardiovascular Disease Research Center, Department of Epidemiology, School of Public Health and Safety, Shahid Beheshti University of Medical Sciences, Tehran, Iran, Tehran, Iran; 4 Department of Surgery, Minimally Invasive Surgery Research Center, Division of Minimally Invasive and Bariatric Surgery, Rasool-e Akram Hospital, Iran University of Medical Sciences, Tehran, Iran; 5 Department of Internal Medicine, School of Medicine, Imam Hossein Hospital, Shahid Beheshti University of Medical Sciences, Tehran, Iran; 6 Student Research Committee, Department of Clinical Pharmacy, School of Pharmacy, Shahid Beheshti University of Medical Sciences, Tehran, Iran; 7 Department of Clinical Pharmacy, School of Pharmacy, Shiraz University of Medical Sciences, Shiraz, 7146864685 Iran

**Keywords:** Obesity, Bariatric surgery, Metabolic dysfunction-associated fatty liver disease, Diabetes mellitus, Longitudinal studies

## Abstract

**Background and aim:**

Metabolic dysfunction-associated fatty liver disease (MAFLD) is highly prevalent, especially in type 2 diabetes mellitus (T2DM). This study evaluates the effect of bariatric surgery on MAFLD in patients with obesity with or without diabetes.

**Methods:**

This registry-based longitudinal cohort study examined the effects of bariatric surgery on MAFLD in patients with obesity (BMI > 30 kg/m^2^) from 2017 to 2023. Clinical data were collected at baseline, 3–6 months, and 9–12 months post-surgery. The primary objective was to assess changes in fatty liver grading.

**Results:**

This longitudinal cohort study, involved 194 patients (100 non-diabetic and 94 diabetic) undergoing bariatric surgery. The mean age was 42.11 ± 11.54 years, with a majority being female (77.84%). The initial mean BMI was 42.42 ± 6.75 kg/m², with non-diabetic patients having a higher mean BMI than diabetic patients. Both groups showed significant decreases in liver enzymes levels post-surgery. Ordinal GEE analysis indicated significant improvements in fatty liver grading for both groups post-surgery. The study found that diabetes, BMI, and triglyceride levels were significantly associated with changes in fatty liver grading.

**Conclusion:**

Our study demonstrates that bariatric surgery leads to improved MAFLD grading within the first year. However, higher BMI and triglyceride (TG) levels post-surgery were linked to poorer outcomes. Additionally, patients with a history of diabetes exhibited a lower likelihood of improvement in MAFLD grading compared to non-diabetic patients. Further clinical trials with larger sample sizes and extended follow-up durations are necessary to substantiate these findings.

## Background

Obesity is a burgeoning global health crisis, closely associated with a spectrum of metabolic disorders, including metabolic dysfunction-associated fatty liver disease (MAFLD) and type 2 diabetes mellitus (T_2_DM). MAFLD, characterized by excessive fat accumulation in the liver, is the most common liver disorder worldwide, affecting approximately 25% of the global population [1]. The disease spectrum ranges from simple steatosis to metabolic dysfunction-associated steatohepatitis (MASH), which can progress to end stage liver diseases [1]. Concurrently, T_2_DM exacerbates the risk of liver fibrosis in patients with MAFLD [2].

Bariatric surgery not only results in significant weight reduction but also reduces risk of liver cirrhosis and even death [3]. In patients with advanced fatty liver disease, bariatric surgery has been associated with an 88% lower risk of progression to end-stage liver diseases including cirrhosis, liver cancer, or liver-related death [3]. Moreover, it has demonstrated a 70% reduction in the risk of developing serious cardiovascular diseases [3].

Bariatric surgery is particularly impactful for patients with diabetes. Studies show that bariatric surgery can induce T2DM remission in 33–90% of individuals one year post-intervention, compared to 0–39% with medical management alone [4]. This highlights the potential of bariatric surgery as a transformative treatment for metabolic disorders in both diabetic and non-diabetic patients, beyond just weight loss [4].

The association between diabetes mellitus and MAFLD is well-documented [5, 6]. MAFLD is highly prevalent in individuals with T_2_DM, affecting up to 70% of this population [5]. These conditions have a bidirectional relationship: diabetes increases MAFLD risk, while MAFLD exacerbates insulin resistance and promotes diabetes progression. MAFLD also increases the risk of cardiovascular disease and hepatocellular carcinoma, making it a significant concern for diabetic patients [5].

This cohort study investigates the effects of bariatric surgery on fatty liver disease in patients with obesity, comparing outcomes in those with and without diabetes. We aim to elucidate the role of bariatric surgery as a MAFLD treatment and its differential impact on patients with and without T2DM. The findings will enhance our understanding of the impact of bariatric surgery on metabolic-related conditions.

## Methods

This registry-based longitudinal (cohort) study was conducted at a private surgical facility under the supervision of Shahid Beheshti University of Medical Sciences, Tehran, Iran, and included patients who underwent bariatric surgery between 2017 and August 2023. The study received ethical approval from the Shahid Beheshti University of Medical Sciences ethics committee (IR.SBMU.RETECH.REC.1402.194). The inclusion criteria were:

- Age 18 years or more.
- Consent to participate in the study.
- Diagnosis of obesity (BMI >30 kg/m²) based on American Diabetes Association (ADA) criteria [7].
- Undergone one of the following bariatric surgeries: Sleeve gastrectomy, Mini-Gastric bypass, and Single Anastomosis Duodenal Ileal Bypass (SADI-S)/Single Anastomosis Stomach–Ileal Bypass with Sleeve Gastrectomy (SASI-S)/Single Anastomosis Sleeve Jejunal Bypass (SASJ).
- Diagnosis of MAFLD:

All patients fulfilled the diagnostic criteria for MAFLD, defined by the presence of hepatic steatosis in addition overweight/obesity, type 2 diabetes, or evidence of metabolic dysregulation [8]. Non-invasive ultrasound imaging was used to assess the hepatic steatosis [9]. The following grading criteria were used:

- Grade I: Diffusely increased hepatic echogenicity, but periportal and diaphragmatic echogenicity is still appreciable.
- Grade II: Diffusely increased hepatic echogenicity obscuring periportal echogenicity, but diaphragmatic echogenicity is still appreciable.
- Grade III: Diffusely increased hepatic echogenicity obscuring periportal as well as diaphragmatic echogenicity [9].

All ultrasound procedures were performed by a single, experienced radiologist to minimize inter-observer variability. Diabetes diagnosis was based on ADA criteria [10].

Data collected included demographics, liver enzyme test (i.e. serum glutamic-oxaloacetic transaminase (SGOT), serum glutamic pyruvic transaminase (SGPT), alkaline phosphatase), lipid profile, fatty liver disease grading and type of bariatric surgery. Clinical data were collected pre-operatively, 3–6 months post-surgery, and 9–12 months post-surgery. A qualified healthcare professional extracted all relevant information from laboratory results and the patient’s medical records.

The primary objective of this study was to assess changes in the grading of fatty liver disease in patients following bariatric surgery throughout the follow-up period. Additionally, changes in the liver enzyme tests were evaluated as secondary endpoint.

### Statistical analysis

The normality of the continuous variables was assessed using histograms. Continuous variables are presented as mean ± standard deviation (SD), and categorical variables as frequency (percentage). Differences between diabetic and non-diabetic patients were assessed using Student’s t-tests or Mann-Whitney U tests, as appropriate based on normality. Equality of variances was assessed using Levene’s test. Differences in categorical variables were assessed using Chi-square or Fisher’s exact tests. Longitudinal changes in laboratory results (lipid profile, liver enzymes, BMI, and MAFLD grade) were analyzed using Generalized Estimating Equations (GEE) with an exchangeable correlation structure to account for repeated measures. Crude and adjusted ordinal GEE models were used to assess factors associated with changes in MAFLD grade. Statistical significance was defined as *p* < 0.05, with 95% confidence intervals (CI). Analyses were performed using STATA version 14.

## Results

In this study from 2017 to 2023, 194 patients (100 non-diabetics and 94 diabetics) underwent bariatric surgery. The mean age of the participants was 42.11 ± 11.54 years, with a majority being female (*n* = 151, 77.84%). The initial mean body mass index (BMI) of the patients was 42.42 ± 6.75 kg/m², with non-diabetic patients exhibiting a statistically higher mean initial BMI compared to their diabetic counterparts (*P* = 0.010).

Among the patients, 145 (74.74%) underwent mini-gastric bypass or gastric bypass procedures, while 27 (13.92%) had sleeve gastrectomy, and 22 (11.34%) underwent SADI-S/SASI-S/SASJ weight loss surgeries. Notably, most patients (51.03%) were classified with fatty liver grade II. A higher proportion of non-diabetic patients were categorized as having grade II and III fatty liver disease compared to diabetic patients, revealing a significant difference in the distribution of fatty liver grading between the two groups (*P* = 0.001). Except for triglyceride levels (*P* = 0.024), no significant differences were observed in other baseline laboratory findings between diabetic and non-diabetic patients (Table 1).

**Table 1:** Basic characteristics of patients underwent bariatric surgery between diabetic and non-diabetics

Variables	Non-diabetic (*n* = 100, 51.55%)	Diabetic (*n* = 94, 48.45%)	Total (*n* = 194)	*p*-value
General information				
Age (years)	37.85 ± 11.49	46.64 ± 9.77	42.11 ± 11.54	< 0.001*
**Gender**				
Female	79 (79.00)	72 (76.60)	151 (77.84)	0.687
Male	21 (21.00)	22 (23.40)	43 (22.16)	
Initial weight (Kg)	116.82 ± 23.03	108.73 ± 19.54	112.90 ± 21.74	0.006*
Height (cm)	163.61 ± 9.12	162.15 ± 8.91	162.90 ± 9.03	0.263
Initial Body Mass Index (BMI, kg/m^2^)	43.48 ± 7.11	41.29 ± 6.18	42.42 ± 6.75	0.010*
**Initial laboratory’s results:**				
SGOT (AST, IU/L)	25.84 ± 14.59	29.28 ± 17.40	27.50 ± 16.06	0.134
SGPT (ALT, IU/L)	33.88 ± 22.72	35.74 ± 19.58	34.77 ± 21.24	0.455
Alkaline phosphatase (IU/L)	181.52 ± 64.004	190.04 ± 68.97	185.66 ± 66.41	0.402
Cholesterol (mg/dl)	189.36 ± 38.56	186.47 ± 46.54	187.97 ± 42.49	0.410
Triglyceride (mg/dl)	162.06 ± 66.25	202.98 ± 117.08	181.75 ± 96.13	0.024*
HDL (mg/dl)	44.77 ± 8.87	44.70 ± 12.96	44.74 ± 10.99	0.620
LDL (mg/dl)	112.65 ± 32.69	106.76 ± 37.80	109.79 ± 35.29	0.110
**Initial grading of hepatic steatosis by Ultrasound Quantification**				
Grade I	3 (3.00)	19 (20.21)	22 (11.34)	0.001*
Grade II	56 (56.00)	43 (45.74)	99 (51.03)	
Grade III	41 (41.00)	32 (34.04)	73 (37.63)	
**Type of bariatric surgery**				
Sleeve gastrectomy	12 (12.00)	15 (15.96)	27 (13.92)	< 0.001*
Mini or Gastric bypass	86 (86.00)	59 (62.77)	145 (74.74)	
SADI-S^1^/SASI-S^2^/SASJ^3^	2 (2.00)	20 (21.28)	22 (11.34)	

Values described as n (%) or mean ± standard deviation

*Statistically significant, *P*-value < 0.05

^1^Single Anastomosis Duodenal Ileal Bypass (SADI-S)

^2^Single Anastomosis Stomach–Ileal Bypass with Sleeve Gastrectomy (SASI-S)

^3^Single Anastomosis Sleeve Jejunal Bypass (SASJ)

**Fig. 1:**
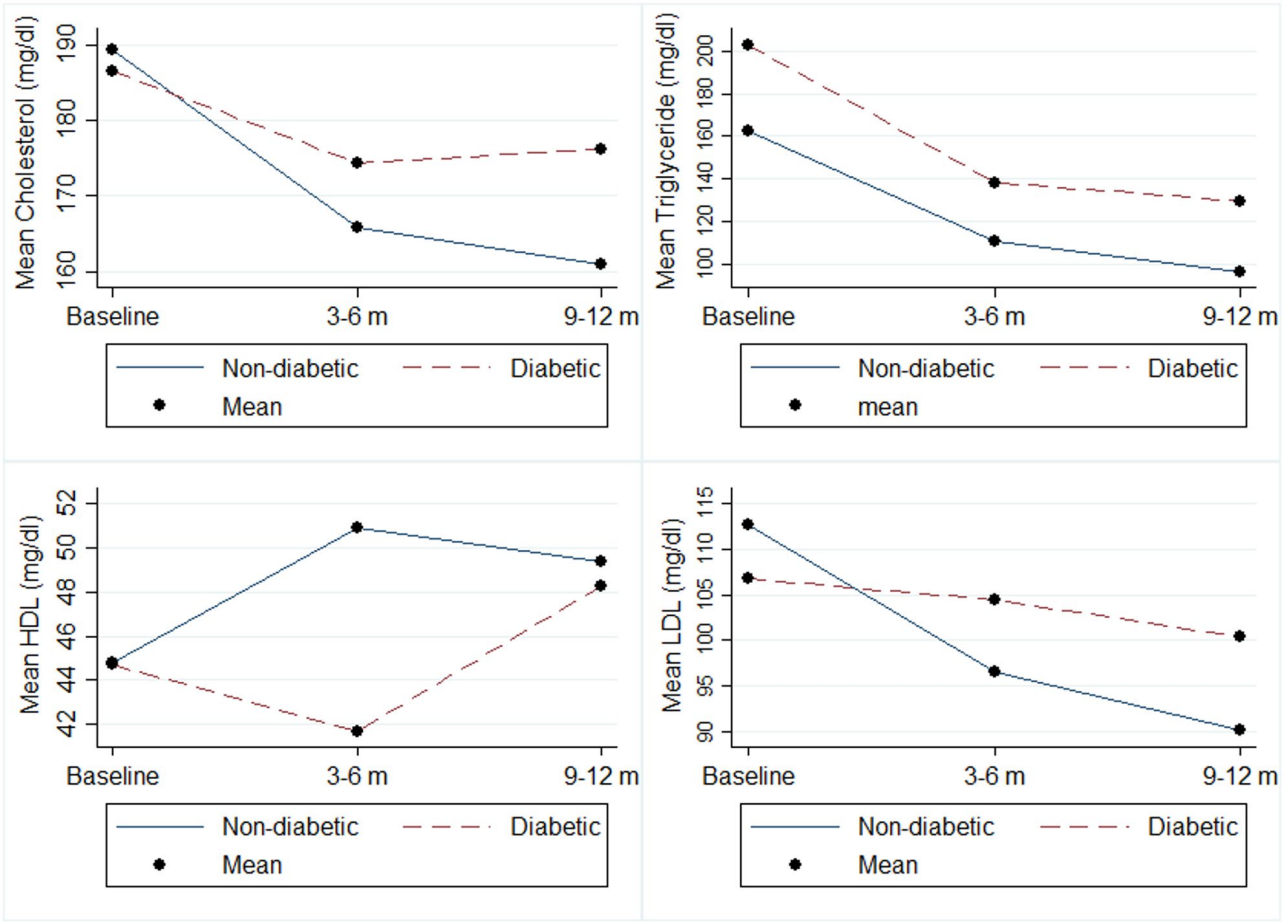
The changes in lipid profile during study period between diabetics and non-diabetics

**Fig. 2:**
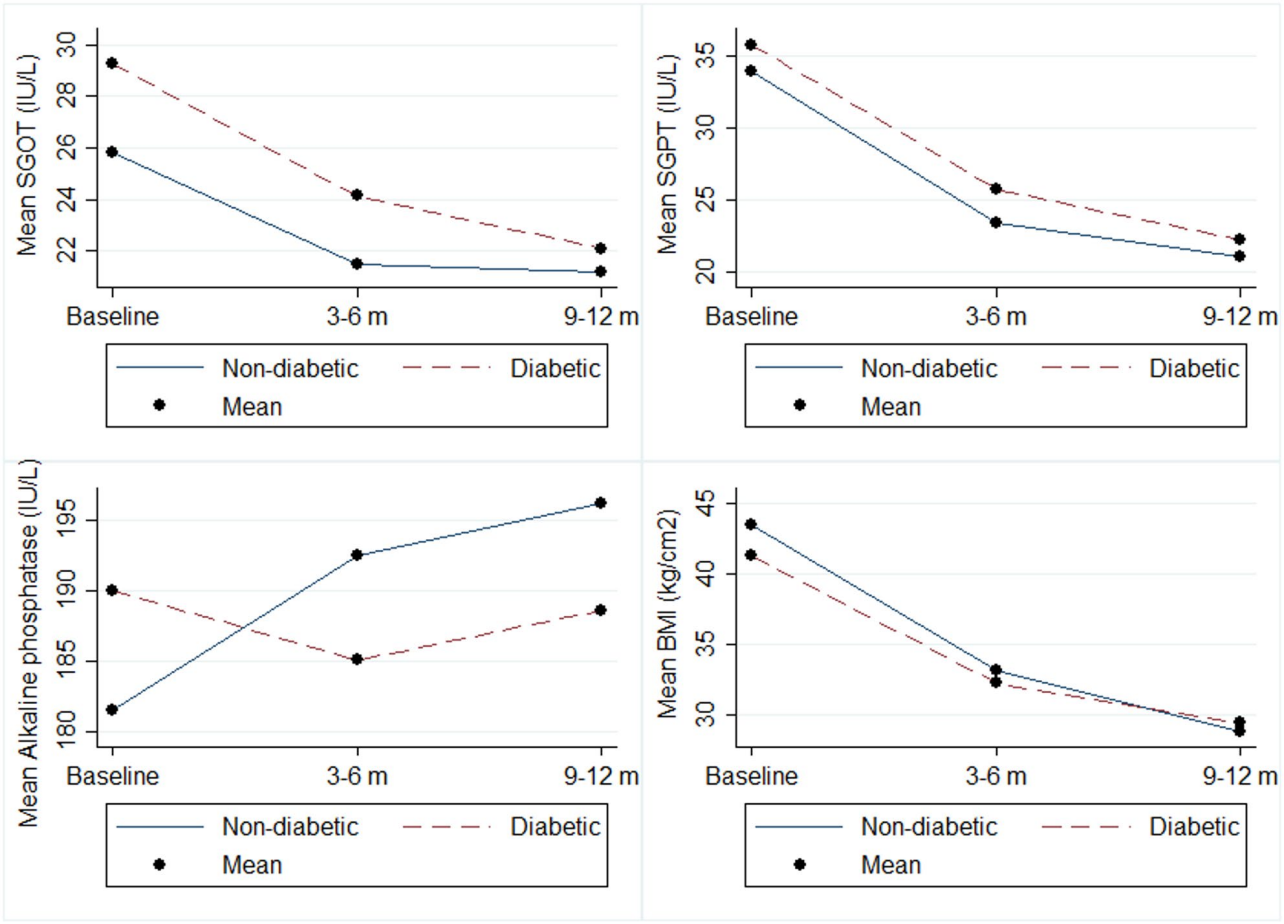
The changes in liver enzymes and body mass index (BMI) during study period between diabetics and non-diabetics

According to the results presented in Table 2, the linear GEE analysis demonstrated a significant decrease in BMI, SGOT, SGPT, and triglyceride levels in both diabetic and non-diabetic patients throughout the study period (*P* < 0.05). Although LDL and total cholesterol levels decreased in both groups, these changes were statistically significant only among non-diabetic patients (*P* < 0.001). Surprisingly, Alkaline phosphatase levels increased significantly in non-diabetic patients during the study period (*P* = 0.029), while the trend for this factor showed a non-significant decrease in diabetic patients (*P* = 0.800). Furthermore, HDL levels exhibited a significant increase in both groups (*P* < 0.001) (Table 2; Figs. 1 and 2).

**Table 2:** Time trend analysis of clinical factors in patients underwent bariatric surgery between diabetic and non-diabetics

Variables	Population	Baseline (T1)	3–6 months (T2)	9–12 months (T3)	*p*-value time effect	*p*-value time × groups	*p*-value Time group comparison
Body Mass Index (BMI, kg/m^2^)	Non-diabetic	43.48 ± 7.11	33.14 ± 5.61	28.82 ± 5.001	< 0.001*	< 0.001*	T1/T2* T1/T3* T2/T3*
	Diabetic	41.29 ± 6.18	32.23 ± 5.46	29.39 ± 4.33	< 0.001*		T1/T2* T1/T3* T2/T3*
SGOT (AST, IU/L)	Non-diabetic	25.84 ± 14.59	21.50 ± 7.56	21.20 ± 6.94	0.003*	< 0.001*	T1/T2* T1/T3*
	Diabetic	29.28 ± 17.40	24.14 ± 11.14	22.10 ± 10.25	0.003*		T1/T2* T1/T3*
SGPT (ALT, IU/L)	Non-diabetic	33.88 ± 22.72	23.37 ± 9.16	21.05 ± 9.05	< 0.001*	< 0.001*	T1/T2* T1/T3*
	Diabetic	35.74 ± 19.58	25.72 ± 15.58	22.22 ± 10.74	< 0.001*		T1/T2* T1/T3*
Alkaline phosphatase (IU/L)	Non-diabetic	181.52 ± 64.004	192.47 ± 62.26	196.19 ± 68.15	0.029*	0.192	T1/T3* T1/T2*
	Diabetic	190.04 ± 68.97	185.04 ± 65.77	188.59 ± 73.79	0.800		Non-significant
Total cholesterol (mg/dl)	Non-diabetic	189.36 ± 38.56	165.78 ± 34.12	161.02 ± 30.66	< 0.001*	< 0.001*	T1/T2* T1/T3*
	Diabetic	186.47 ± 46.54	174.38 ± 44.80	176.25 ± 36.71	0.072		T1/T2* T1/T3*
Triglyceride (mg/dl)	Non-diabetic	162.06 ± 66.25	110.83 ± 33.49	96.15 ± 49.38	< 0.001*	< 0.001*	T1/T2* T1/T3* T2/T3*
	Diabetic	202.98 ± 117.08	138.20 ± 45.73	129.53 ± 53.49	< 0.001*		T1/T2* T1/T3*
HDL (mg/dl)	Non-diabetic	44.77 ± 8.87	50.91 ± 17.37	49.39 ± 10.21	< 0.001*	< 0.001*	T1/T2* T1/T3*
	Diabetic	44.70 ± 12.96	41.69 ± 9.55	48.28 ± 12.05	< 0.001*		T1/T3* T2/T3*
LDL (mg/dl)	Non-diabetic	112.65 ± 32.69	96.46 ± 30.55	90.13 ± 24.78	< 0.001*	< 0.001*	T1/T2* T1/T3*
	Diabetic	106.76 ± 37.80	104.45 ± 35.20	100.40 ± 29.58	0.298		Non-significant
Fatty liver grading	Non-diabetic						
	Normal	0 (0.0)	40 (40.82)	62 (75.61)	< 0.001*	< 0.001*	T1/T2* T1/T3* T2/T3*
	Grade I	3 (3.00)	39 (39.80)	20 (24.39)			
	Grade II	56 (56.00)	18 (18.37)	0 (0.0)			
	Grade III	41 (41.00)	1 (1.02)	0 (0.0)			
	Diabetic						
	Normal	0 (0.0)	5 (5.68)	12 (36.36)	< 0.001*		T1/T2* T1/T3* T2/T3*
	Grade I	19 (20.21)	54 (61.36)	18 (54.55)			
	Grade II	43 (45.74)	25 (28.41)	3 (9.09)			
	Grade III	32 (34.04)	4 (4.55)	0 (0.0)			

Values described as mean ± standard deviation or frequency and percentage

* Statistically significant, P-value < 0.05 based on Generalized Estimation Equation (GEE) method

The ordinal GEE analysis indicated a statistically significant improvement in fatty liver grading for both groups (*P* < 0.001). Notably, 9 to 12 months after bariatric surgery, the prevalence of fatty liver grades II and III in non-diabetic patients was zero. Similarly, during this period, the prevalence of fatty liver grade II decreased to 9.09% in diabetic patients, while the prevalence of fatty liver grade III reduced to zero (Table 2).

After assessing the interaction effects for each factor, we observed a significant effect between time and patient groups (diabetic and non-diabetic) (*P*_time×groups_ < 0.05), with the exception of alkaline phosphatase levels (*P*_time×groups_ = 0.192). This indicates that the impact of bariatric surgery on clinical parameters varied between diabetic and non-diabetic patients throughout the study period. Further details of the time trend analysis are presented in Table 2, as well as Figs. 1 and 2.

Based on the results of Table 3, univariate ordinal GEE analysis showed that the type of bariatric surgery, history of diabetes, mean BMI, and mean triglyceride levels during the study were significantly associated with changes in fatty liver grading (*p* < 0.05). Additionally, after considering the effects of potential confounders in a multivariable ordinal GEE model, it was observed that in diabetics compared to non-diabetics, the odds of remaining in fatty liver grade III during the study period were 81% higher in the diabetic group compared to the non-diabetic group (OR = 1.81, 95% CI = 1.07, 3.06, *P* = 0.025). An increase of one unit in the mean BMI during the study was associated with 20% increased odds of remaining in fatty liver grade III (OR = 1.20, 95% CI = 1.16, 1.24, *P* < 0.001). Furthermore, an increase of one unit in the mean triglyceride levels during the study was associated with a slight increase in the odds of remaining in fatty liver grade III (OR = 1.007, 95% CI = 1.003, 1.01, *P* < 0.001) (Table 3).

**Table 3:** Results of univariate and multivariable ordinal generalized estimating equation (GEE) analysis about associated factors in changes of fatty liver grading

Variables	Crude OR (95% CI)	p_value	Adjusted OR (95% CI)	p_value
**Gender**				
Female	Reference	0.117	Reference	0.577
Male	1.31 (0.93, 1.85)		1.15 (0.69,1.91)	
Age (years)	1.006 (0.99, 1.01)	0.264	0.99 (0.97,1.01)	0.482
**Type of bariatric surgery**				
Sleeve gastrectomy	Reference	-	Reference	-
Mini or Gastric bypass	0.61 (0.41, 0.90)	0.014*	0.61 (0.31,1.17)	0.136
SADI-S^1^/SASI-S^2^/SASJ^3^	1.24 (0.61, 2.51)	0.553	0.64 (0.28,1.46)	0.291
**Diabetes**				
No	Reference	0.001*	Reference	0.025*
Yes	1.66 (1.23, 2.25)		1.81 (1.07, 3.06)	
Mean of BMI during follow-up (kg/m ^2^ )	1.25 (1.21, 1.30)	< 0.001*	1.20 (1.16, 1.24)	< 0.001*
Mean of Triglyceride (mg/dl) during follow-up	1.01 (1.006, 1.01)	< 0.001*	1.007 (1.003, 1.01)	< 0.001*

Odds ratio, 95% Confidence Interval

^*^Statistically significant,* P-*value < 0.05

^1^ Single Anastomosis Duodenal Ileal Bypass (SADI-S)

^2^ Single Anastomosis Stomach–Ileal Bypass with Sleeve Gastrectomy (SASI-S)

^3^ Single Anastomosis Sleeve Jejunal Bypass (SASJ)

## Discussion

In this study, we explored the impact of different types of bariatric surgery on the status of fatty liver disease in patients with obesity. Our investigation shed light on key findings. By analyzing data collected over the study period, we uncovered that this type of surgery is associated with resolving of the fatty liver in most of the patients after one year. This finding is in line with the previously performed studies [11–13].

Also, in our study patients who did not suffer from diabetes may have better chance of the disease resolution. It would be because of the altered metabolism which is more prominent in patients with diabetes [14].

In a multicenter, randomized trial conducted in Italy, to compare bariatric surgery with lifestyle interventions and medical therapy for non-alcoholic steatohepatitis (NASH). Participants were assigned to one of three groups: lifestyle modification plus medical care, gastric bypass, or sleeve gastrectomy. It was shown that both surgical groups (gastric bypass and sleeve gastrectomy) significantly outperformed lifestyle modification. Bariatric-metabolic surgery was more effective in treating NASH, with no reported deaths or life-threatening complications [15].

Furthermore, we have found no significant associated of the type of the surgery and the outcome of the patients (i.e. improving in grading of the fatty liver disease). Data from previously performed studies are not conclusive for the amount of weight loss and its correlation to patient’s better outcome regarding the fatty liver disease. Although no significant correlation was found between the amount of weight loss and MAFLD outcome after bariatric surgery [16], But, we identified that if the patients experience higher BMI and TG levels after surgery, it would be associated with worse control of fatty liver disease. In Abangah et al. study, it was shown that BMI and TG levels are correlated with the severity of fatty liver disease based on the findings on ultrasonography [17]. And Cazzo et al. demonstrated that, the BMI of the patients of the bariatric surgery after the procedure is significantly associated with resolution of the fibrosis [18]. Also, as we stated before, history of diabetes is an important factor correlates with worse outcome.

The SPLENDOR study which was conducted in patients with obesity and NASH, substantial and sustained weight loss achieved with bariatric surgery can protect the patients. Among patients with nonalcoholic steatohepatitis and obesity, bariatric surgery, compared with nonsurgical management, was associated with a significantly lower risk of incident major adverse liver outcomes and major adverse cardiovascular events [3]. This is very important. Many patients especially ones with underlying conditions including diabetes and hypertension whom are at increased risk of cardiovascular diseases and events may benefit from bariatric surgery.

Another study which evaluates the long-term effect of bariatric surgery in patients with NASH, it was shown that the surgery is associated with resolution of NASH at one year after the surgery. Also, the authors stated that this favorable outcome will continue for 5 years after surgery which is remarkable [19]. It is in line with our findings. In one year follow up we observed that many patients experienced resolution of the disease which is an important factor. It should be said that we did not follow the included patients after 1 year.

The unexpected increase in ALP levels observed in non-diabetic patients may reflect not only hepatobiliary complications such as gallstone formation but also alterations in bone metabolism and increased bone turnover following rapid weight loss, as reported in post-bariatric surgery cohorts [20].

In our study, hepatic steatosis was assessed using ultrasound, which remains one of the most widely available and non-invasive imaging modalities in routine clinical practice. While advanced modalities such as transient elastography or liver biopsy provide more definitive assessment of steatosis and fibrosis, their limited availability and invasiveness restrict their use in large-scale, registry-based studies. Therefore, although ultrasound offers practical feasibility, the absence of fibrosis evaluation and the potential underestimation of steatosis severity represent important limitations of our work that should be considered when extrapolating the results. Ultrasound demonstrates reasonable accuracy for detecting moderate to severe fatty liver, but its sensitivity is notably reduced in patients with obesity, limiting its reliability compared to histological assessment, specifically in our study [9, 21, 22]. Most previous studies in this field have utilized biopsy-proven NASH, which provides greater methodological strength compared to our study [15, 19, 23]. Because it is an invasive and expensive procedure, we did not perform liver biopsy of the patients. Also, we recommend that a clinical trial investigating the effect of bariatric surgery on MAFLD in patients with or without diabetes should be conducted.

Our study provides evidence of short-term improvements in steatosis following bariatric surgery; however, other limitations should be acknowledged. The absence of platelet count data prevented calculation of non-invasive fibrosis scores (i.e. FIB-4, NFS, or APRI), thereby limiting evaluation of disease progression beyond steatosis. Fibrosis is the most important predictor of long-term liver-related morbidity and mortality in MAFLD and previously performed studies included this and demonstrated that bariatric surgery could improve fibrosis in this population [3].

A further limitation of our study is the relatively short follow-up duration of 9–12 months. While our findings demonstrate early improvements in metabolic status and liver histology following bariatric surgery, the durability of these changes remains uncertain. Previous long-term studies, such as that of Lassailly et al., have shown that regression of fibrosis is often a gradual process that may require several years to become evident [19]. Therefore, our conclusions are restricted to short-term outcomes, and future investigations with extended follow-up are warranted to determine whether the observed benefits persist or progress over time.

Because of the small sample size (*n* = 22), SADI-S, SASI-S, and SASJ procedures were grouped together, which may obscure procedure-specific effects given their distinct malabsorptive mechanisms; therefore, our findings should be interpreted with caution.

Finally, the grouping of distinct surgical procedures with small sample sizes may have obscured procedure-specific effects. These limitations highlight the need for future prospective studies with comprehensive laboratory data, longer follow-up, and advanced imaging or histological assessment to better define the impact of bariatric surgery on MASLD outcomes.

In conclusion, our study demonstrates improvement in MAFLD grading post-bariatric surgery during the first year. Higher BMI and TG levels after surgery were associated with worse outcome. Also, Patients with the history of diabetes have lower chance of improvement in MAFLD grading compared to non-diabetic patients. More studies specifically clinical trials with larger sample size and longer follow up duration are needed.

## Data Availability

The data supporting the results of this study are available upon request. Patients’ personal information and identifiable data were not shared.

## Abbreviations

MAFLD: Metabolic dysfunction-associated fatty liver disease
T2DM: Type 2 diabetes mellitus
MASH: Metabolic dysfunction-associated steatohepatitis
SADI-S: Single Anastomosis Duodenal Ileal Bypass
SASI-S: Single Anastomosis Stomach–Ileal Bypass with Sleeve Gastrectomy
SASJ: Single Anastomosis Sleeve Jejunal Bypass
SGOT: Serum glutamic-oxaloacetic transaminase
SGPT: Serum glutamic pyruvic transaminase
SD: Standard deviation
GEE: Generalized Estimating Equations
CI: Confidence intercvals
NASH: Non-alcoholic steatohepatitis
